# Adenosquamous carcinoma of the pancreas: a case report

**DOI:** 10.1186/1757-1626-3-41

**Published:** 2010-02-01

**Authors:** Evangelia Skafida, Xanthippi Grammatoglou, Chryssoula Glava, Dimitrios Zissis, Nikolaos Paschalidis, Eleftheria Katsamagkou, Nikolaos Firfiris, Thivi Vasilakaki

**Affiliations:** 1 Department of Pathology, "Tzaneion" General Hospital of Piraeus, Zanni & Afentouli 1, Piraeus-Greece; 2 Department of Gastroenterology, "Tzaneion" General Hospital of Piraeus, Zanni & Afentouli 1, Piraeus-Greece; 3 Department of General Surgery, "Tzaneion" General Hospital of Piraeus, Zanni & Afentouli 1, Piraeus-Greece; 4 Department of Anesthesiology, General Hospital of Larissa, Tsakalof 1, Larissa-Greece

## Abstract

Adenosquamous carcinoma of the pancreas is a rare variant of pancreatic exocrine carcinoma. We report a case of 70 year old man who came to our hospital with abdominal pain, anorexia and jaundice. Imaging of the abdomen showed a mass in the region of the head of the pancreas. Histological evaluation of the pancreatic tumor showed an adenosquamous carcinoma which was extensively infiltrative with perineural invasion, involvement of peripancreatic lymph nodes and all the thickness of the duodenum wall. The tumor exhibited a biphasic malignant growth identified as well to moderate differentiated adenocarcinoma and well to poorly differentiated squamous cell carcinoma.

## Introduction

Adenosquamous carcinoma of the pancreas is a rare aggressive subtype of pancreatic ductal adenocarcinoma and accounts for 1-4% of all exorcine malignancies of the pancreas.

Also referred to as mucoepidermoid carcinoma and adenoacanthoma in older literature, pancreatic adenosquamous carcinoma is characterized by adenomatous cell population mixed with varying amounts of keratinized squamous cell elements [1-3] The prognosis of this rare lesion appears to be even less favorable than the invasive ductal tumour, with only a few patients surviving more than 1 year and none of the therapies seem to prolong survival in this aggressive lesion [1-6].

## Case presentation

**Figure 1:**
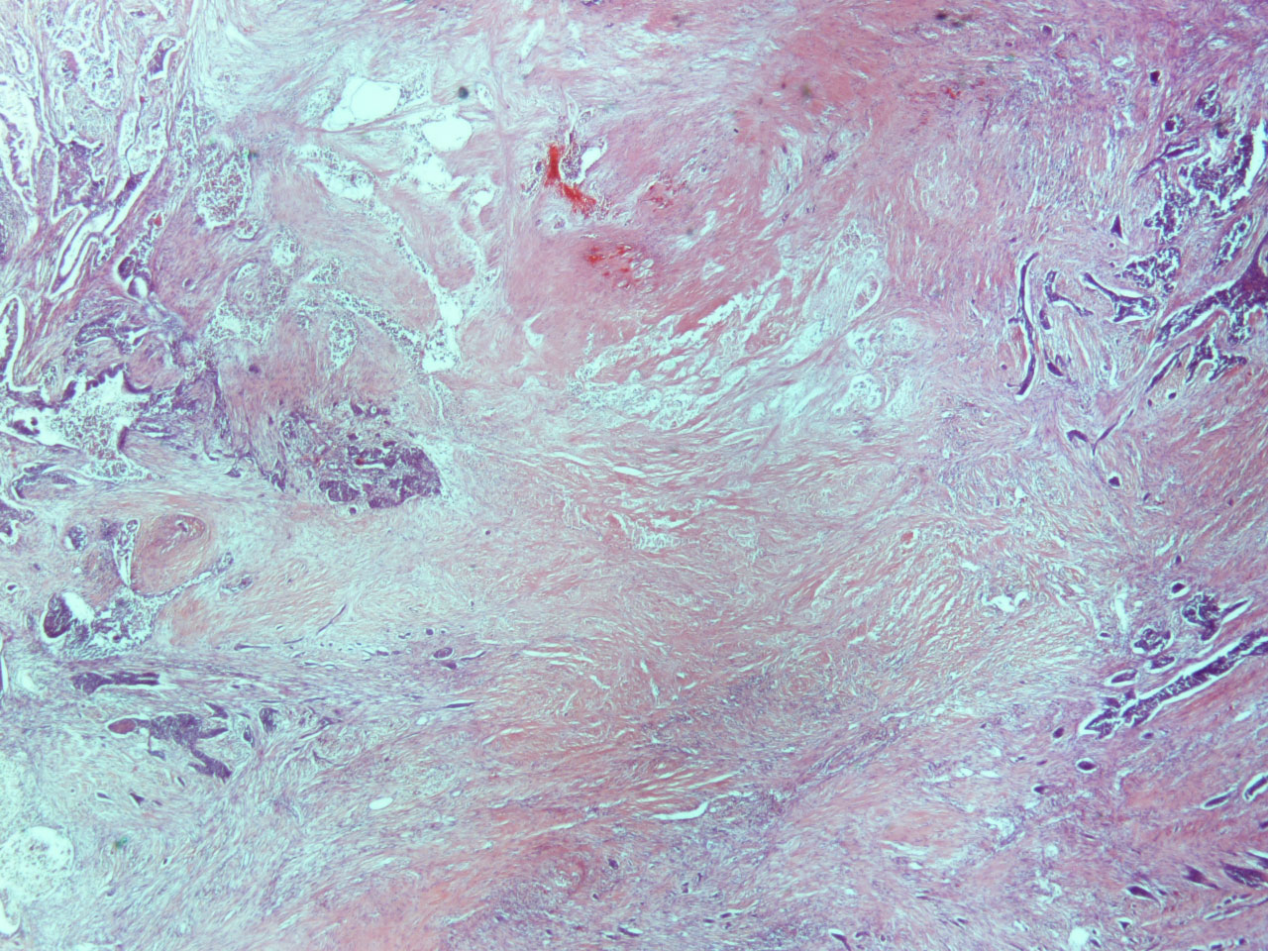
**Adenosquamous carcinoma of the pancreas with area of necrosis (H&E × 20)**.

**Figure 2:**
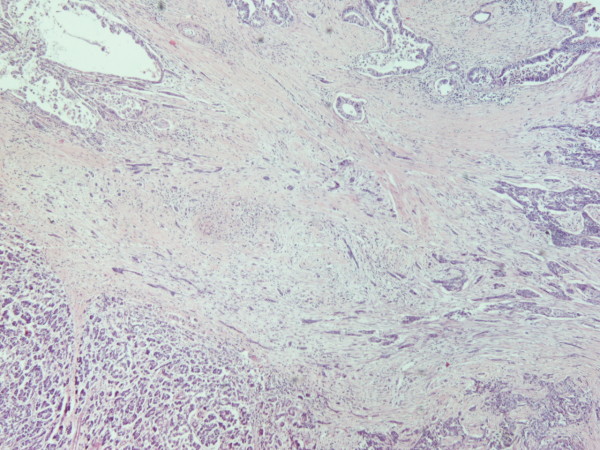
**Adenosquamous carcinoma of the pancreas (H&E × 40)**.

**Figure 3:**
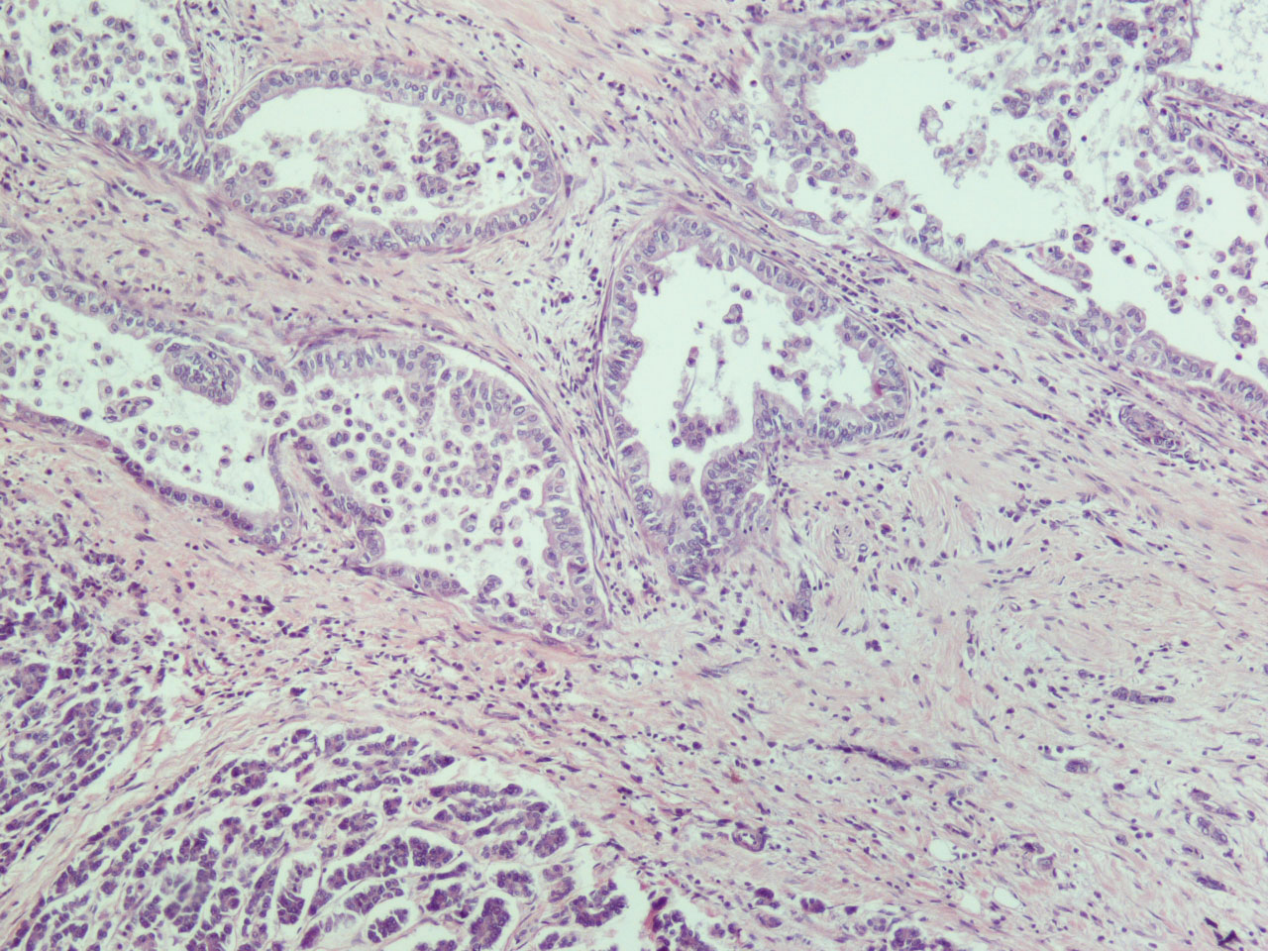
**Adenocarcinoma similar to ductal carcinoma (H&E × 100)**.

**Figure 4:**
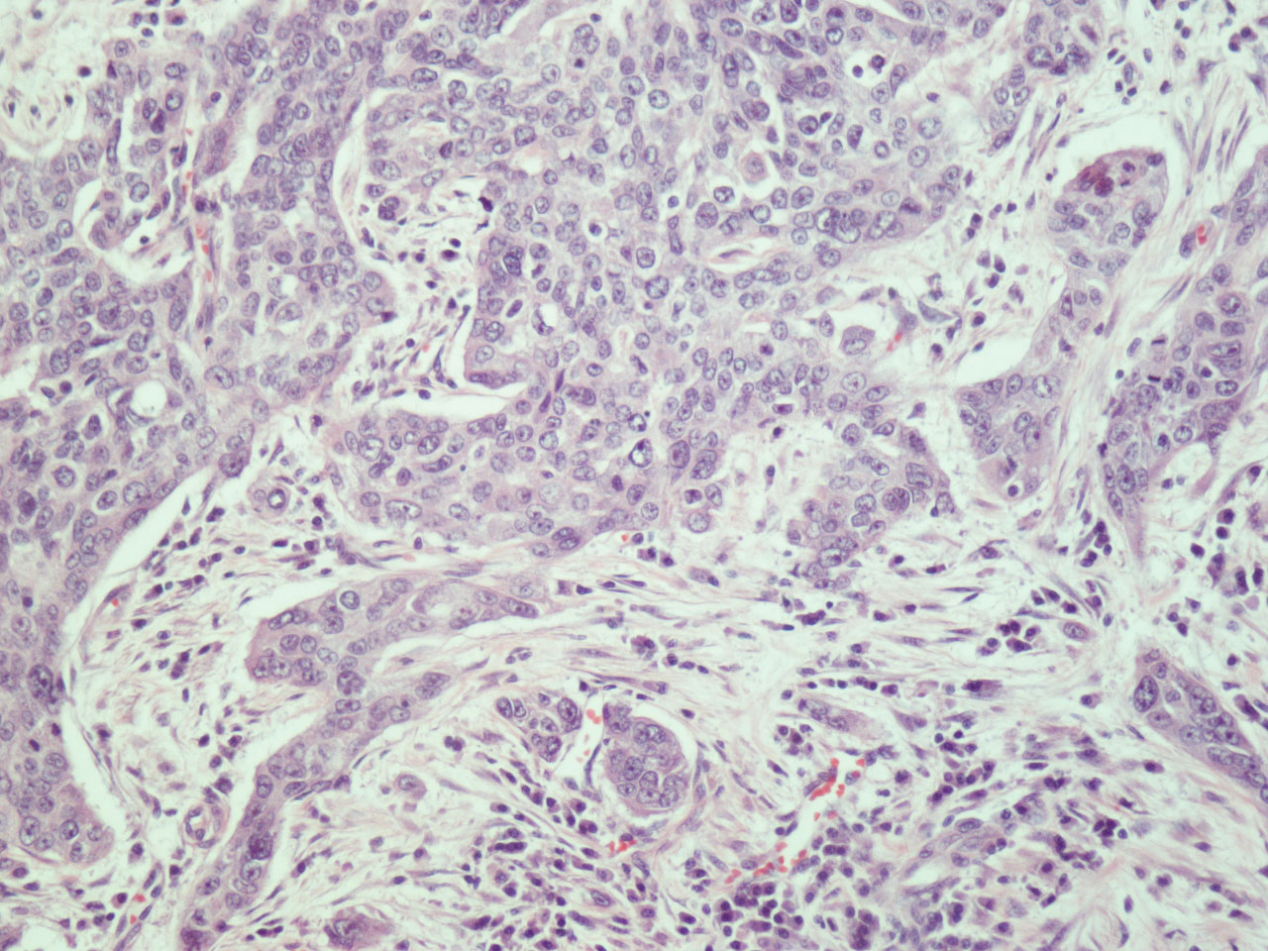
**Squamous cell carcinoma composed of irregular nests of polygonal cells with no gland formation (H&E × 200)**.

**Figure 5:**
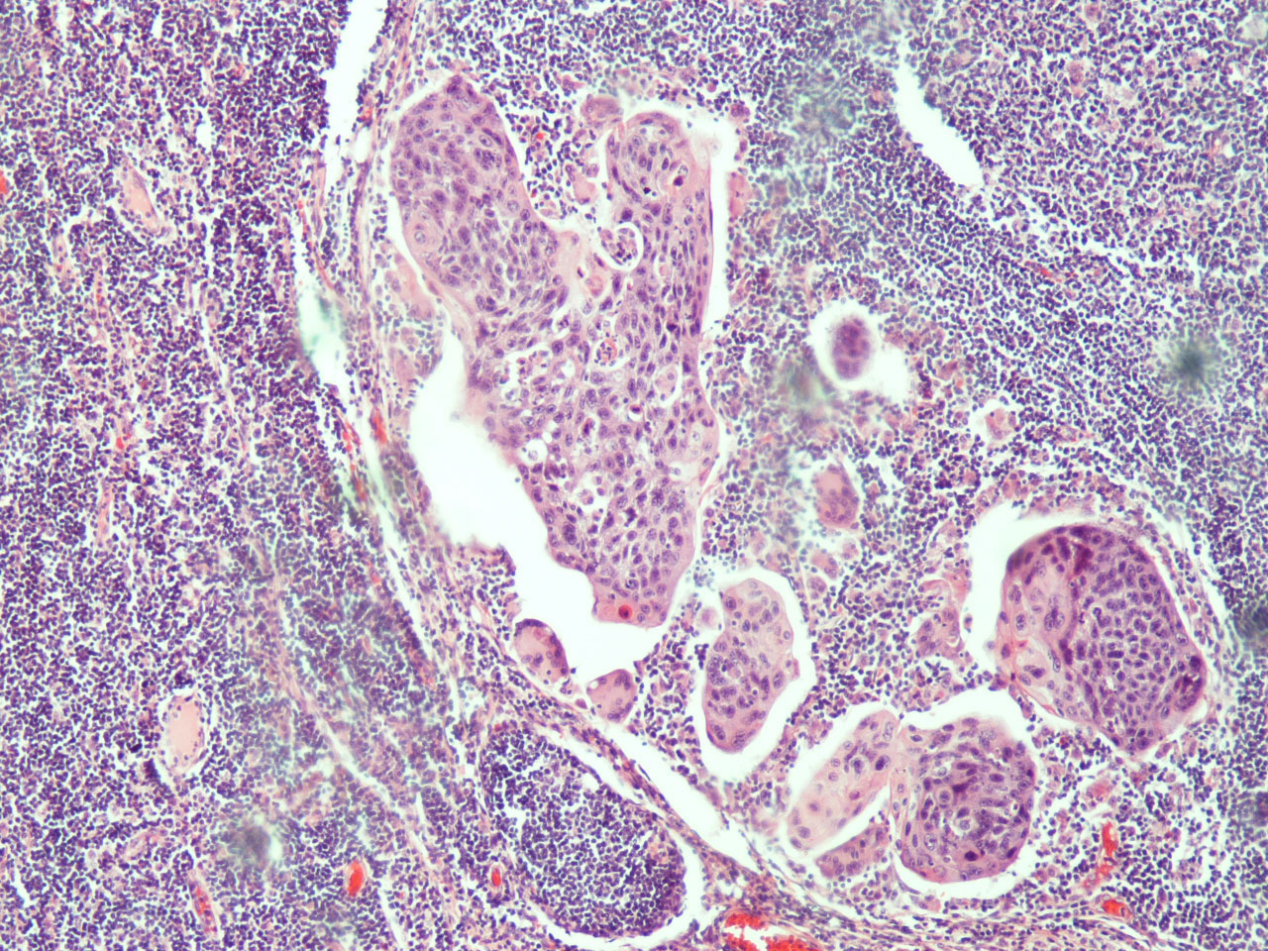
**Lymph node metastasis only from the squamous element (H&E × 100)**.

**Figure 6:**
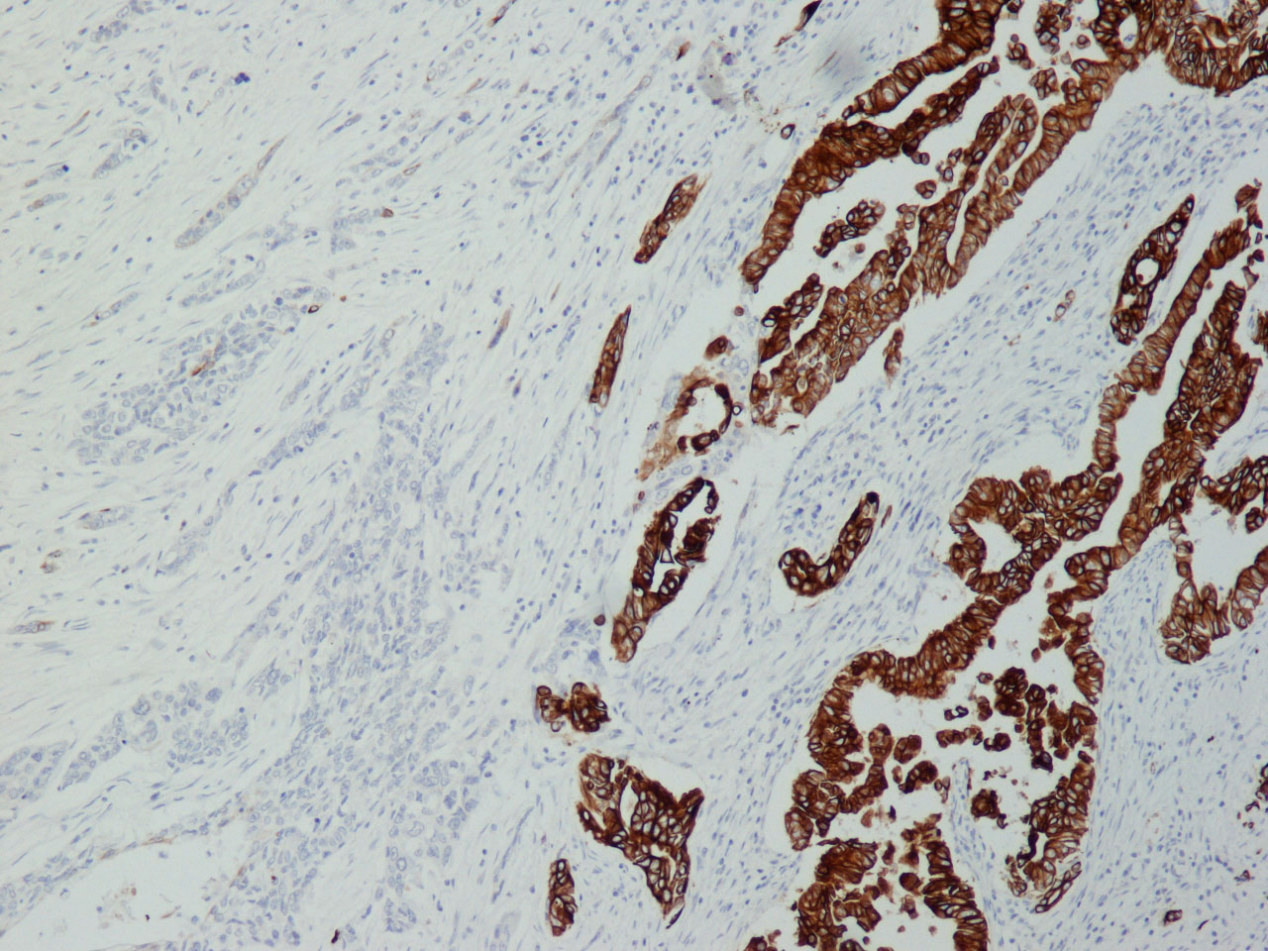
**Ker7 showed predilection for adenocarcinoma (Ker 7 × 100)**.

**Figure 7:**
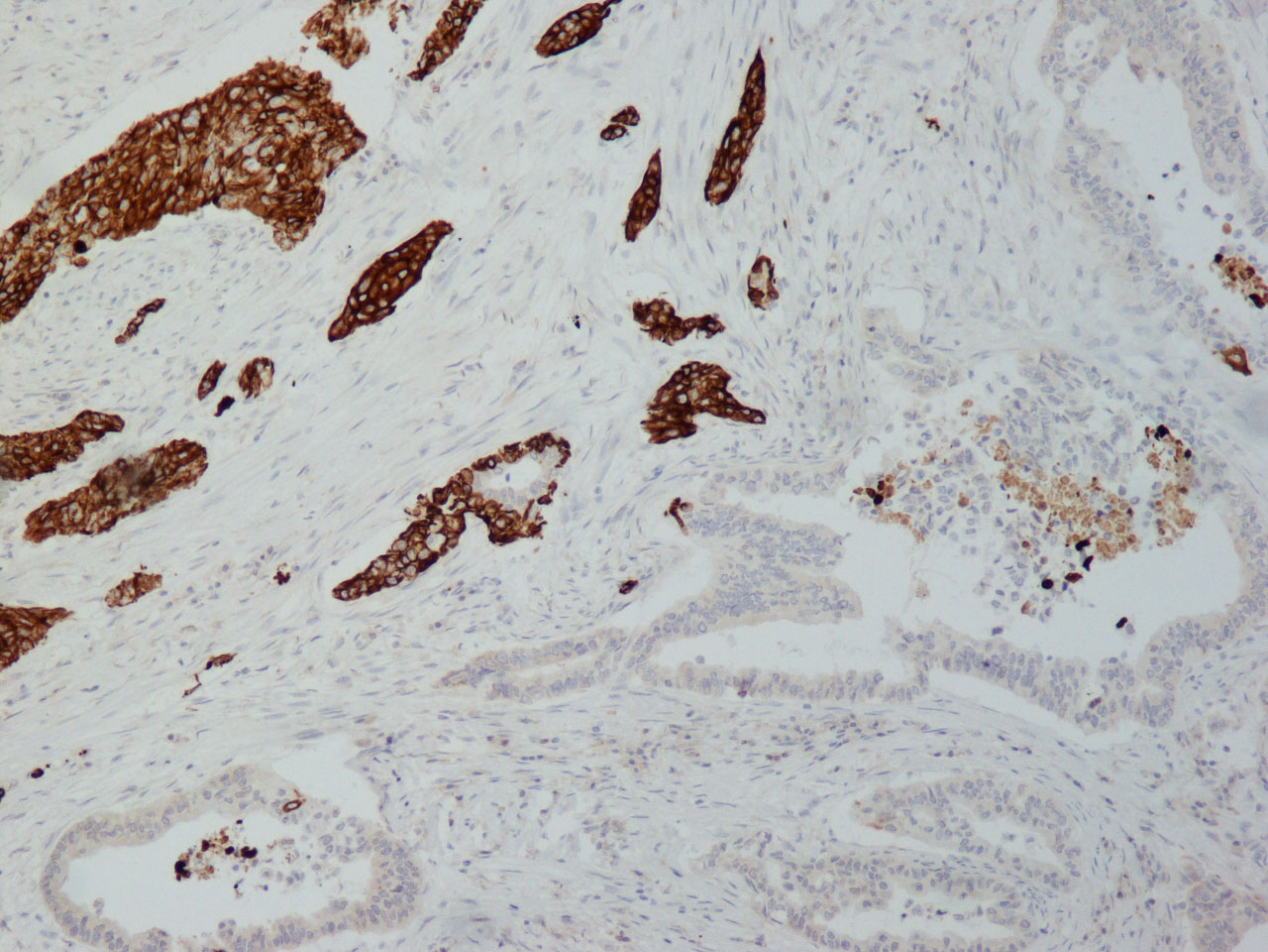
**The squamous component was strongly immunoreactive with CK5/6, while the glandular component was negative (CK5/6 × 100)**.

A 70 year old man from Greece having Hellenic Nationality, came to the emergency department of our hospital with abdominal pain, anorexia and jaundice. The duration of symptoms prior to presentation was four weeks. Physical examination revealed moderate tenderness on palpation of the hypogastrium. His past medical history included essential hypertension and hyperlipidemia. There was no family history of gastrointestinal diseases. Laboratory studies showed elevated bilirubin and alkaline phosphatase levels and moderately elevated serum carbohydrate antigen 19-9 (Ca 19-9) of 380 U/ml (reference value ≤ 70 Uml). Abdominal ultrasound and computerized tomography scan showed a mass in the head of the pancreas measuring 4.5 cm in size and curative pancreaticoduodenectomy was performed. Histological evaluation of the pancreatic tumor showed an adenosquamous carcinoma which was extensively infiltrative with perineural invasion, involvement of peripancreatic lymph nodes and all the thickness of the duodenum wall. The tumor elicited an intense desmoplastic stromal reaction and areas of necrosis (Figure 1). Focal areas of high grade pancreatic intraepithelial neoplasia were seen. The tumor exhibited a biphasic malignant growth identified as well to moderate differentiated adenocarcinoma and well to poorly differentiated squamous cell carcinoma (Figure 2). The adenocarcinoma component contained ductal or glandular structures with focal intracellular or extracellular mucin (Figure 3). Squamous differentiation was characterized by irregular and infiltrative nests or sheets of polygonal cells with distinct cellular borders, intercellular bridges, eosinophilic cytoplasm and varying degrees of keratinization (Figure 4). These two different patterns could be seen separated topographically within the substance of the tumor or intimately admixed. Six of the 15 resected lymph nodes were positive for metastatic tumor which was composed of squamous carcinoma only (Figure 5). The immunochistochemical study showed that the tumor cells were positive for cytokeratin AE1 and AE2. Cam 5.2 and Ker 7 were reactive predominantly in the adenocarcinoma component and in few squamous cells (Figure 6). Immunoreactivity for CK 5/6 was restricted to the squamous component, while the glandular component was negative (Figure 7). A few number of tumor cells were immunoreactive with CEA and Ca 19-9. All tumor cells were negative for Ker 20, chromogranin and synaptophysin. The patient received postoperative adjuvant chemotherapy and he is alive 6 months after surgery.

## Discussion

Pancreatic adenosquamous carcinoma is a rare aggressive subtype of ductal adenocarcinoma and accounts for 1-4% of all exocrine malignancies of the pancreas. Also referred to as mucoepidermoid carcinoma and adenoacanthoma in older literature, adenosquamous carcinoma demonstrates both malignant squamous cell and glandular differentiation [1,2]. Squamous metaplasia of the pancreatic ductal epithelium occurs most commonly in the setting of chronic pancreatitis, but is noted in the adjacent ducts of only about 4% of adenocarcinomas [2]. Squamous metaplasia of pre-existing adenocarcinoma has been suggested by some authors as a mechanism underlying the histogenesis of pancreatic adenosquamous carcinoma. Furthermore, it has been suggested in the literature that squamous cell carcinoma occurs de novo within the pancreas without an identifiable ductal component. There are several theories, none well proven, about the origin of the adenosquamous tumour. Primary pure squamous cell carcinoma of the pancreas is rare and the presence of only malignant squamous cells in an aspirate from a pancreatic mass should almost always point to a diagnosis of adenosquamous carcinoma rather than a primary squamous cell carcinoma. Making a diagnosis of adenosquamous carcinoma on fine needle aspiration before surgery can be difficult and it is possible when aspirates show evidence of both squamous and glandular differentiation, although one component often predominates and features of dual differentiation may be focal [1,6]. It is worth noting that adenosquamous carcinoma also may represent a metastasis to the pancreas arising from lung, colon, esophagus, salivary glands and female reproductive organs. Most pure squamous lesions of the pancreas are thought to represent metastatic disease [1,2,6]. The first known report of adenosquamous carcinoma in the literature is credited to Herxheimer in 1907 in which he referred to this lesion as "cancroide". Kardon et al published the largest series of adenosquamous carcinomas of the pancreas. The reviewed 25 cases all of which showed biphasic malignant components. The two types of carcinoma were present in variable proportions in their series [6]. Most metastases reported in the literature were from the adenocarcinomatous component and not from the squamous component [6]. In our case, however, the metastatic deposits were all from the squamous component. Immunophenotypically the glandular and squamous cell areas were distinctive. Keratin expression differed with the adenocarcinoma reacting with anti - CK 7 and the squamous cell carcinoma reacting with anti - CK 5/6 [2,7]. This differential antigen expression should not be construed as proof of separate clonality or origin but rather as different phenotypic expression within a single neoplastic proliferation [2]. The major symptoms are no different than those of the usual invasive ductal lesions of the pancreas and include abdominal pain, weight loss, anorexia, malaise, nausea, fatigue, pruritus and jaundice. Abdominal pain is the most common presenting symptom followed by anorexia, weight loss and jaundice [1,2,8]. The prognosis of adenosquamous carcinoma appears to be even less favorable than the invasive ductal tumour, with only a few patients surviving more than 1 year. The use of adjunctive chemotherapy or radiation may increase the duration of survival. However, owing to the rarity of adenosquamous carcinoma, the number of cases is too small to statistically support this claim [2,4,5,8-10].

## Consent

Written informed consent was obtained from the patient for publication of this case report and accompanying images. A copy of the written consent is available for review by the Editor-in-Chief of this journal

## Competing interests

The authors declare that they have no competing interests.

## Authors' contributions

ES drafted the article. XG contributed to the writing and revising of the manuscript. CG performed the histological examination. DZ analyzed and interpreted the patient data. NP analyzed and interpreted the patient data. EK and NF participated in its design. TV supervised the preparation of the article and helped in final manuscript. All authors read and approved the final manuscript.
